# Achievement of Clinical Remission in Life-Threatening Rosai-Dorfman Disease With Cladribine

**DOI:** 10.7759/cureus.74576

**Published:** 2024-11-27

**Authors:** Lex P Leonhardt, Hiroshi Yamagata, John Harcha, Alejandro Calvo

**Affiliations:** 1 Hematology and Medical Oncology, Kettering Health, Kettering, USA

**Keywords:** cladribine, extranodal sinus histiocytosis with massive lymphadenopathy, pericardial tamponde, pleural effusions, rosai–dorfman disease

## Abstract

Rosai-Dorfman disease (RDD) is a rare proliferative histiocytic disorder characterized by sinus histiocytosis with massive lymphadenopathy, rarely presenting with severe and life-threatening extra-nodal features. The rarity of RDD, clinically variant phenotype, limited data, and lack of a current standardized management approach make treatment decisions difficult. Herein, we present a case of life-threatening, disseminated RDD with rare clinical features of recurrent pericardial effusion, bilateral pleural effusions, and abdominal tissue fibrosis successfully treated with six cycles of cladribine, achieving clinical remission. Compilation of positive outcomes is required to establish a uniform treatment approach.

## Introduction

Rosai-Dorfman disease (RDD) is a rare sinus histiocytosis with massive lymphadenopathy (SHML) of unknown etiology, formerly reported by Juan Rosai and Ronald Dorfman in 1969 [1]. Histiocytes in RDD characteristically show immunohistochemical findings of S-100+, CD68+, and CD1a-, associated with frequent occurrence of emperipolesis [2]. Although a majority of patients with RDD manifest with benign and self-limited cervical lymphadenopathy, 43% of patients with RDD present with extra-nodal involvement, often associated with poor outcomes [3]. Extra-nodal involvement most commonly occurs in the skin, soft tissues of the head and neck, paranasal sinuses, upper respiratory tract, and bone, with rare reports of intrathoracic (2%), cardiac (0.1 to 0.2%) and gastrointestinal involvement (less than 1%) [3-5]. Although the first consensus multidisciplinary recommendations for managing RDD were reported in 2018, no uniform approach exists. Mixed results have been reported with various chemotherapies (cladiribine, vinca alkaloids, CHOP, methotrexate/6-mercaptopurine), immunomodulators (lenalidomide, thalidomide, rituximab), and tyrosine kinase inhibitors (imatinib, cobimetinib) [2]. While positive results have been reported with varying drugs in each medication class, the primary challenge remains the lack of data supporting a standard approach due to the rarity and heterogeneous nature of RDD. Herein, we present a case of life-threatening RDD with rare clinical features of recurrent pericardial effusion, bilateral pleural effusions, and abdominal tissue fibrosis successfully treated with cladribine.

## Case presentation

**Figure 1:**
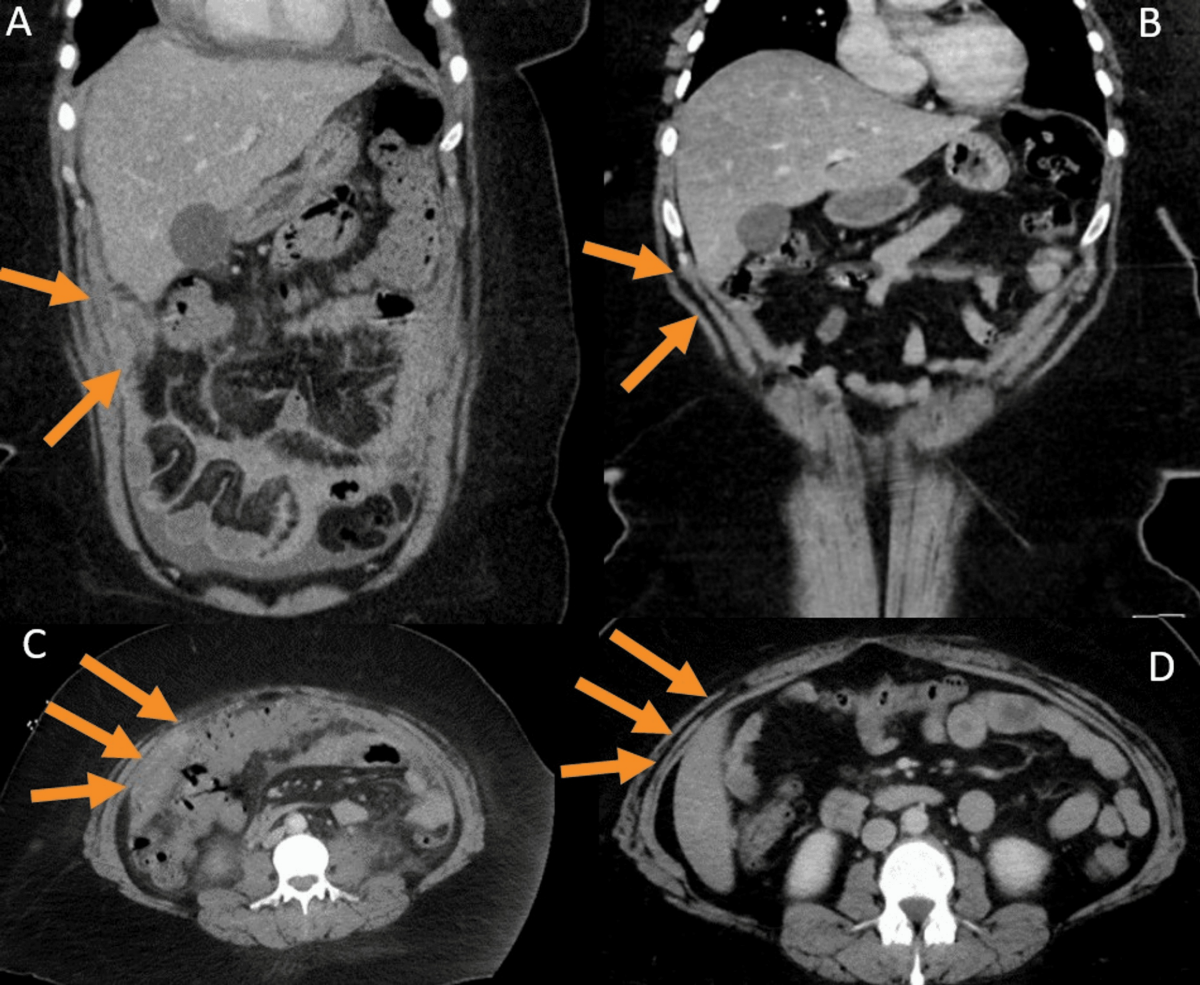
Subtle omental nodularity before (left column, images A & C) and after six cycles of cladribine therapy (right column, images B & D).

A 40-year-old healthy Hispanic female presented to our emergency department with abdominal pain, constipation, and dyspnea. A contrasted CT chest demonstrated a moderate pericardial effusion and large bilateral pleural effusions with pleural thickening and nodular lesions in the anterior mediastinum. Ultrasound-guided left thoracentesis with 1200 cc pleural fluid evacuation was performed, consistent with exudate by Light’s criteria. Pleural fluid cytology revealed inflammatory cells only. Contrasted CT abdomen/pelvis showed diffuse peritoneal thickening and greater omental nodularity with similar findings on MRI abdomen/pelvis (Figure 1A, 1C). CT-guided omental biopsy was negative for malignancy, showing inflammatory cells only.

**Figure 2:**
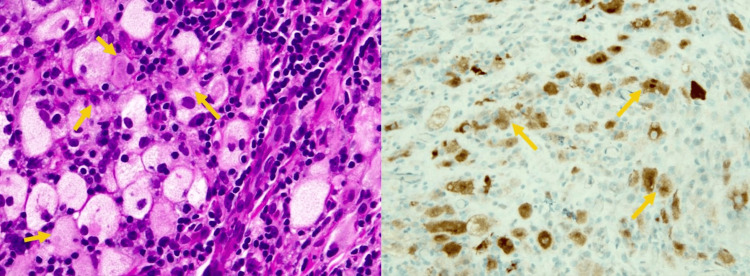
Left image showing immunohistochemical staining of pleural tissue showing proliferation of histiocytes and evidence of emperipolesis (H&E, original magnification 400x)and right imaging showing immunohistochemical staining of pleural tissue demonstrating S-100 positivity (S100, original magnification 200x).

The patient developed recurrent bilateral pleural effusions, requiring five subsequent therapeutic thoracenteses in the following two months. Serial cytology was negative for malignancy with redemonstration of inflammatory cells by cytology. Given recurrent abdominal pain, exploratory laparoscopy with peritoneal and omental biopsies was performed. Pathology demonstrated fibrous tissue with inflammatory infiltrate. Left video-assisted thoracoscopy with pleural biopsies and mechanical pleurodesis was performed with high intraoperative suspicion for lymphoma or mesothelioma. The frozen section of pleural nodule sampling was concerned with malignancy. Immunostaining of pleural tissue with CD3 and CD20 showed an admixture of T-lymphocytes and B-lymphocytes. CD68 highlighted abundant histiocytes. An outside expert pathologic review reported lymphohistiocytic infiltration of the pleura, demonstrating emperipolesis of lymphocytes and plasma cells (Figure 2, left). Staining with S-100 stain showed multiple foci of positivity in the histiocytes (Figure 2, right).

**Figure 3:**
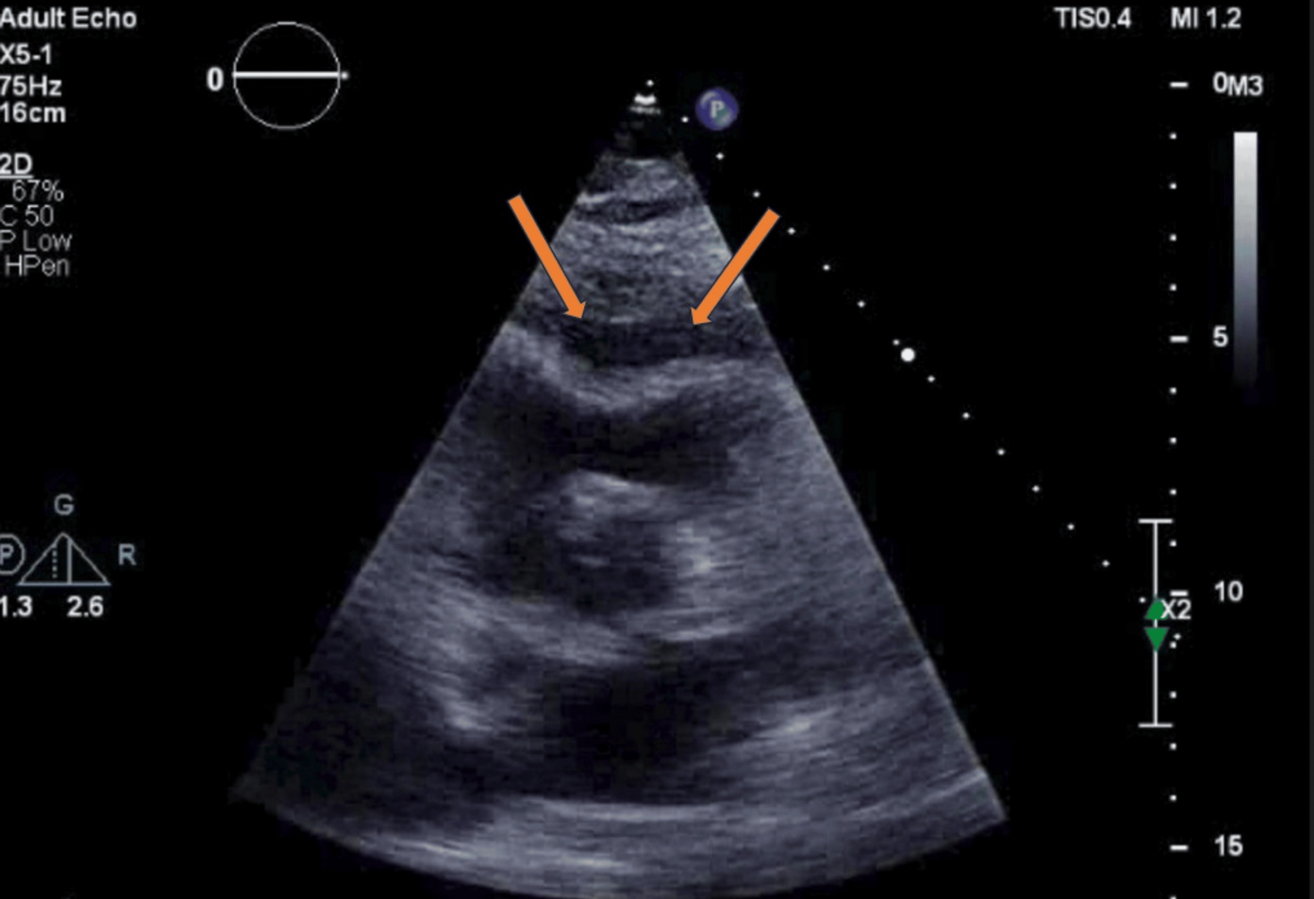
2D echocardiogram demonstrating early right ventricular diastolic collapse, consistent with early cardiac tamponade physiology.

These findings were consistent with a diagnosis of RDD. BRAF stain was negative. One month later, the patient rapidly decompensated with evidence of cardiac tamponade requiring an emergent pericardial window (Figure 3).

**Figure 4:**
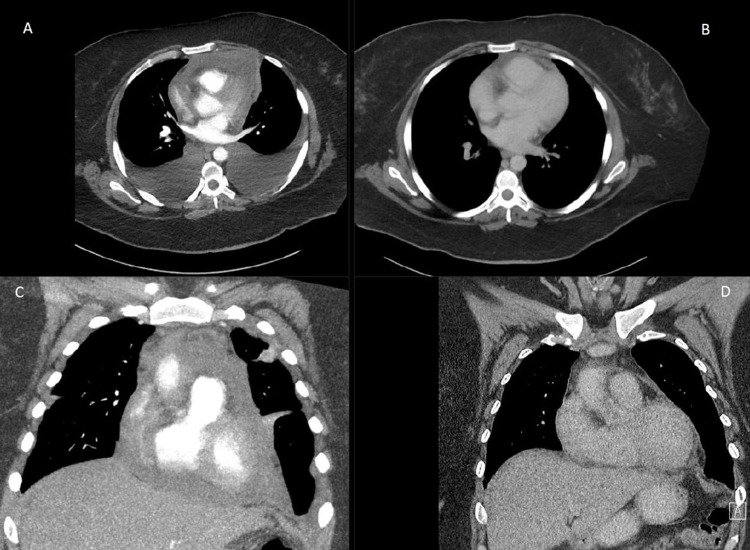
Computed tomography chest with intravenous contrast prior to initiation of Cladribine (left column, images A & C) compared to contrasted CT chest imaging after six cycles of cladribine (right column, images B & D), demonstrating complete radiographic resolution of pericardial and pleural effusions.

She developed high oxygen requirements and unstable hemodynamics with evidence of massive pleural effusions (Figure 4A, 4C). Medical oncology recommended cladribine 5 mg/m^2^ on days 1-5 every 28 days. Rapid clinical improvement was noted after the initiation of cladribine, and the patient was discharged home after completing one cycle of therapy. She continued outpatient cladribine with a resolution of pulmonary and abdominal symptoms. After three cycles, repeat CT imaging showed radiographic improvement with decreasing size of pleural effusions, pericardial effusion, and omental disease. After six cycles, restaging CT chest, abdomen, and pelvis showed resolution of cardiopulmonary and peritoneal disease, consistent with clinical and radiographic complete remission (Figure 1B, 1D and Figure 4B, 4D).

The patient tolerated cladribine without significant adverse effects. Tissue next-generation sequencing (NGS) was attempted but unsuccessful due to insufficient tissue samples. The patient continues exhibiting complete clinical and radiographic remission four years from the initial diagnosis.

## Discussion

The rarity and clinically variant phenotype of RDD limits treatment options to case-specific circumstances, with no uniform treatment proposal. The first consensus multidisciplinary recommendation for diagnosis and management of RDD was published in June 2018 [2]. One of the current recommendations for severe, disseminated, or refractory disease is cladribine 5mg/m^2^ intravenously daily for five days every 28 days for six cycles [2]. Prior to this recommendation, only small retrospective case series and reports had been described, demonstrating variable success [2,6-10]. A retrospective observational study performed at MD Anderson in 2019 reported an 80% response rate and median progression-free survival of 29 months in five patients with RDD treated with cladribine from 1995 to 2015 [9]. The rapid response and durable complete remission demonstrated in our case strongly support the use of cladribine in patients with high-risk extra-nodal features and organ compromise. While the exact mechanism of action of purine analogs in RDD is unclear, the rapid onset cytotoxic effect of cladribine in patients with visceral crisis and end-organ damage should be strongly considered when making therapeutic decisions, whereas more indolent forms of RDD may be better served by observation or less toxic systemic therapy. Compilation of additional case reports and small studies is required to develop standard treatment recommendations.

A retrospective chart review reported cladribine as second-line therapy in six patients with refractory RDD from 1994 to 2017, demonstrating a 67% overall response rate after three to four cycles with no relapse at a 16-month follow-up [10]. Additional case reports confirm similar findings with variable duration of cladribine therapy, ranging from three to six cycles [2,7-10]. Our case demonstrated a durable clinical response to six cycles in the setting of severe, refractory, life-threatening multi-system disease. Despite recent consensus guidelines, the duration of cladribine therapy is inconsistent in current literature. Variability in treatment duration is likely attributed to clinician experience in treating RDD, unpredictable clinical response to chemotherapy, and toxicities of cladribine. With the accumulation of data over time, a more uniform approach will hopefully be established.

Cladribine is a purine nucleoside analogue, activated to inhibit DNA synthesis and repair, approved for use in hairy cell leukemia and relapsing multiple sclerosis, with off-label indications in AML, mantle cell lymphoma, and Waldenstrom’s macroglobulinemia [10]. The efficacy of cladribine in RDD is felt to be related to its ability to impair monocyte function through interleukin-6 (IL-6), interleukin-1B (IL-1B), and tumor necrosis factor-alpha (TNF-a) inhibition [2,8]. This is supported by a previous report describing the normalization of IL-6 and TNF-a after treatment with cladribine, suggesting the benefit of monitoring pro-inflammatory cytokines to assess response to therapy [8]. The most common side effects of cladribine include myelosuppression, fevers, severe anemia, and fatigue [11]. Given the toxicity profile, this treatment modality is often reserved for refractory or life-threatening RDD. Our patient did not report any clinically significant adverse effects to cladribine.

In the past decade, BRAF V600E mutations have been reported in Erdheim Chester disease (ECD) and LCH, a significant advancement with treatment implications in these closely related diseases. BRAF V600E mutations have not been reported in RDD thus far, although KRAS and MAP2K1 mutations have been identified in common mitogen-activated protein kinase/extracellular signal-related kinase pathways (MAPK/ERK) in a small subset of RDD patients [12]. This supports previous theories of clonal etiology in a subset of RDD patients, which could be promising for the use of targeted therapies. Additionally, NRAS, KRAS, MAP2K1, and ARAF mutations have been identified in another small group of RDD patients, again raising the question of clonal origin in some forms of RDD. MEK-1 inhibition has shown a preliminary response in one patient with KRAS-mutated RDD [2]. While the cytoreductive properties of cladribine are well established in RDD, further investigation into inhibition of pro-inflammatory cytokines (TNF-a, IL-6, IL-1B) and effect on molecular targets (NRAS, KRAS, MAP2K1, ARAF) may be useful to fully understand the mechanism of response and develop future targeted therapies.

NGS was reportedly limited by the lack of adequate tissue in our patient, but it should be considered in all patients presenting with extra-nodal RDD requiring treatment. It will be acquired in our patient if there is evidence of relapse. Liquid biopsy may be a consideration in a subset of patients as well, although the dynamics and tumor fraction of circulating tumor DNA in RDD are unclear. The current success of targeted therapy in ECD and LCH is encouraging, especially with evidence of targetable mutations in MAPK/ERK pathways in certain subsets of patients with RDD. Further investigation is required to establish these correlations before considering targeted therapy in RDD.

## Conclusions

Overall, while cladribine is not universally effective for all patients with RDD, it offers a valuable treatment option, especially for those with severe or refractory forms of the disease. Further research and clinical trials are needed to better understand its role and optimize its use in the management of RDD.
